# Nrf2 Participates in M2 Polarization by *Trichinella spiralis* to Alleviate TNBS-Induced Colitis in Mice

**DOI:** 10.3389/fimmu.2021.698494

**Published:** 2021-06-23

**Authors:** Xuemin Jin, Xue Bai, Ying Zhao, Zijian Dong, Jianda Pang, Mingyuan Liu, Xiaolei Liu

**Affiliations:** ^1^ Key Laboratory of Zoonosis Research, Ministry of Education, Institute of Zoonosis, College of Veterinary Medicine, Jilin University, Changchun, China; ^2^ Department of Nephrology, First Hospital of Jilin University, Changchun, China; ^3^ Jiangsu Co-Innovation Center for Prevention and Control of Important Animal Infectious Diseases and Zoonoses, Yangzhou, China

**Keywords:** *Trichinella spiralis*, macrophage, TNBS, colitis, Th2, Nrf2

## Abstract

*Trichinella spiralis* induced alternative activated macrophages (M2), leading to protect against Crohn’s disease, known as Th1 –related inflammation, which enhances oxidative stress in the host. However, the relationship of oxidative stress and *T. spiralis* –mediated immune response is still unknown. In our study, we showed that nuclear factor erythroid 2-related factor-2 (Nrf2), a key transcription factor in antioxidant, participated in M2 polarization induced by *T. spiralis* muscle larval excretory/secretory (ES) products *in vitro*. ES –treated M2 were injected intravenously after TNBS challenge and we demonstrated that ES-M could alleviate the severity of the colitis in mice. Adoptive transfer of ES –treated M2 decreased the level of IFN-γ and increased the levels of IL-4 and IL-10 *in vivo*. However, the capacity of ES –treated Nrf2 KO macrophages to treat colitis was dramatically impaired. ES –treated Nrf2 KO macrophages was insufficient to result in the elevated levels of IL-4 and IL-10. These findings indicate that Nrf2 was required for M2 polarization induced by *T. spiralis* ES to alleviate colitis in mice.

## Introduction

Crohn’s disease (CD) is a chronic relapsing inflammatory condition of the gastrointestinal tract with increased production of Th1 cells (1). Countries where helminth infection are endemic have a lower incidence of CD than non-endemic countries, and there have been interesting reports of the beneficial effects of helminths in CD patients (2), indicating that the regulatory effect of eliminating helminths often leads to imbalances in the immune system and increases immune-mediated diseases (3). Several studies in animals and clinical trials provide strong evidence that helminth can downregulate CD –specific immune responses (3). Unfortunately, this kind of therapy is hard to accept for patients because of the fear of helminths.

Macrophages play crucial roles in immune responses and are the main target in the treatment of CD. Helminths alleviate colitis through polarization of alternatively activated macrophages (M2) (4), which mediate Th2 type responses, contributing to suppression of Th1 type response (5). Recently, cellular immunotherapy is suggested to be a novel therapeutic option to downregluate colitis –related immune response and inflammation (6–8). *Trichinella spiralis* muscle larval excretory/secretory (MLES) products induce M2, which can ameliorate inflammation of colitis (9). Macrophage-based therapy offers hope for the development of a safe, effective and viable treatment for inflammatory diseases in humans (10). However, the mechanism of helminth –induced macrophages remain unclear.

Despite tremendous efforts, the etiology of CD remains unclear. It has been generally accepted that CD is related to strong oxidative stress and is an important factor in causing colon inflammation (11). The nuclear factor (erythroid-derived 2)-like-2 factor (Nrf2) is a key regulatory transcription factor in the regulation of antioxidant response element–dependent genes (12). Induction of Nrf2 pathway decreases uncontrolled inflammation such as colitis (13). It has been reported that activation of Nrf2 leads to M2 macrophage polarization (14, 15). Recent study have demonstrated that upregulated Nrf2 expression contributes to immnunomudulatory role of *T. spiralis* infection in host (16), however, details related to the mechanism is still unknown.

In our study, we aimed to investigate the role of Nrf2 in the development of macrophages polarized by *T. spiralis*. *In vitro*, we found that murine bone marrow-derived macrophages were activated to M2 phenotype by ES of *T. spiralis* in Nrf2 dependent manner. We demonstrated that ES -M2 had a therapeutic effect on 2,4,6-trinitrobenzene sulfonic acid (TNBS)-induced CD model in mice. ES –treated M2 could downregulate the Th1-related inflammation and upregulate the Th2 immune response *in vivo*. However, the capacity of ES –treated Nrf2 KO macrophages to treat colitis was dramatically impaired. Our results provided a new evidence of ES –treated M2, which may lead to a potentially new approach in the treatment of CD and revealed that Nrf2 participated in the development of ES –treated M2.

## Materials and Methods

### Animals and Ethics Statement

BALB/c mice (female, 6–8 weeks old) and C57JBL/6 mice (female, 6–8 weeks old), and Wistar rats (female, 6–8 weeks old) were purchased from the Experimental Animal Centre of College of Basic Medical Sciences, Jilin University (Changchun, China). Nrf2 knockout (KO) C57BL/6 mice were purchased from the Jackson Laboratory (Bar Harbor, ME, USA) and kept in a temperature-controlled room (22 ± 2°C) under a 12 h dark–light cycle. All animal experiments were performed according to regulations of the Administration of Affairs Concerning Experimental Animals in China. The protocol was approved by the Institutional Animal Care and Use Committee of Jilin University (20170318) (8).

### *T. spiralis* and Preparation of ES

The *T. spiralis* isolate (ISS534) was obtained from a naturally infected domestic pig in Henan Province in China. Preparation of muscle larvae ES was obtained as described previously (17). Wistar rats were orally infected with 3000 infective larvae, and *T. spiralis* muscle larvae were recovered at 35 days post infection (dpi). All *T. spiralis* muscle larvae were washed and incubated separately in prewarmed serum-free RPMI 1640 medium containing 2 mM l-glutamine, 100 U/ml penicillin, and 100 μg/ml streptomycin at 37°C under 5% atmospheric CO_2_ for 24 h. After centrifugation, the supernatant containing ES products was dialyzed and concentrated. According to the manufacturer’s instructions, the endotoxin was removed from the protein by using the ToxOut™ High Capacity Endotoxin Removal kit (Biovision, USA). There was about approximately 0.134 EU/ml residual endotoxin existing in MLES, approximately equivalent to 20 pg/mg endotoxin (17, 18). The protein concentration was quantified with a BCA protein assay kit (Thermo Scientific). We made three different preparations of ES products from muscle larvae of *T. spiralis* to perform several independent experiments. One representative experiment is shown here.

### Bone Marrow-Derived Macrophages (BMDM) Isolation, Culture, and Stimulation

**Figure 1:**
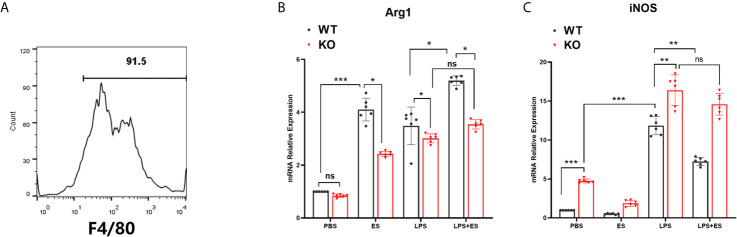
Expression of iNOS and Arg1 in wild type (WT) or Nrf2 KO macrophages induced by *T. spiralis* ES. Macrophages were enriched by positive selection with anti- F4/80 magnetic beads. The enriched F4/80^+^ macrophages were typically of > 90% purity as determined by flow cytometry **(A)**. Total RNA was extracted from cells and mRNA expression levels of Arg1 **(B)** and iNOS **(C)** were quantified using RT-PCR. The results are presented as the mean ± SD of each group (n=6) *P < 0.05, **P < 0.01, ***P < 0.001, ns, no significance, as indicated by the line (one-way ANOVA with Tukey’s *post hoc* test).

Bone marrow-derived macrophages (BMDM) were generated from murine bone marrow cells as described previously (19). Briefly, bone marrow cells were obtained from wild type (WT) and Nrf2 KO C57JBL/6 mice and cultured in DMEM medium containing growth factors of 20 ng/ml recombinant M-CSF (Sigma–Aldrich) and 10% FBS at 37°C, 5% CO_2_. The culture medium was replaced every 72 h. Adherent cells were collected after 6 days whereas non-adherent cells were removed by washing the plates with phosphate buffer solution (PBS) twice. Macrophages were enriched by positive selection with anti- F4/80 magnetic beads (Miltenyi Biotec). The enriched F4/80^+^ macrophages were typically of > 90% purity as determined by flow cytometry (**Figure 1A**). To determine the phenotype of macrophages, macrophages were stimulated with sterile PBS, ES or LPS alone for 24 h or macrophages were pre-treated with ES (50 μg/ml) for 24 h before stimulation with LPS (100 ng/ml, Sigma–Aldrich) for 24 h. Cell culture supernatants were collected and stored at −80°C for the following experiments.

### RNA Extraction and Real Time (RT)-PCR

mRNA expression levels were quantified using RT-PCR as described previously (8). Briefly, RNA from 5×10^6^ cells from each sample was extracted with Trizol reagent (Invitrogen) according to the manufacturer’s instructions. Complementary DNA (cDNA) was synthesized with a reverse transcriptase kit (Vazyme, Nanjing, China). RT-PCR was performed using SYBR Green QPCR Master Mix (TaKaRa, Japan). The primers used for RT-PCR are listed in **Table 1**.

**Table 1:** The primers of quantitative RT-PCR.

Genes	Primer	Sequence(5′→3′)
**iNOS**	Forward primer	AAAGTGACCTGAAAGAGGAAAAGGA
	Reverse primer	TTGGTGACTCTTAGGGTCATCTTGTA
**Arg-1**	Forward primer	CTCCAAGCCAAAGTCCTTAGAG
	Reverse primer	AGGAGCTGTCATTAGGGACATC
**GAPDH**	Forward primer	ACTCCACTCACGGCAAATTC
	Reverse primer	TCTCCATGGTGGTGAAGACA

### Establishment of Colitis Model for Adoptive Transfer of Macrophages in Mice

Colitis was generated by intrarectal administration of 2,4,6-trinitrobenzene sulfonic acid (TNBS) solution (sigma, USA) as described previously (8). Briefly, BALB/c mice were fasted for 24 h with free access to drinking water and then were anesthetized using sodium pentobarbital (50 mg/kg, ip). Next, a catheter was inserted through the anus to approximately the level of the splenic flexure and the colon was then infused with mixture (5% TNBS and absolute ethyl ethanol with ratio 1:1) intrarectally. The mice in control group were administrated with 50% ethyl ethanol in the same way. The mice were allowed to eat and drink ad libitum from 1 h after the operation. Mice were randomly divided into 4 groups of six mice each. For adoptive transfer, WT and Nrf2 KO macrophages stimulated with ES were washed (x3) with sterile PBS, and 1 × 10^6^ cells in 500 μL of sterile PBS were injected intravenously (i.v.) immediately after TNBS challenge. Three days later, mice were humanely euthanized by CO_2,_ and then the colon and spleen were collected for the following experiments. The effect of macrophages on TNBS –induced colitis was evaluated in three independent experiments.

### Assessment of Colitis

The mice in each group were observed daily and given a clinical disease score (disease activity index, DAI) ranging from 0 to 12 (**Table 2**). DAI was assessed by body weight loss, stool consistency, and stool bleeding, which were all recorded every-day as described previously (8). Approximately 1 cm of colon was resected for histopathology examination, fixed in 4% neutral-buffered formalin, embedded in paraffin, sectioned at 5µm thickness and stained separately with hematoxylin and eosin (H&E), according to standard protocols as described previously (8). The histological damage scoring was based on the following 2 parameters: epithelial lesion (0, none damage; 1, some loss of goblet cells; 2, extensive loss of goblet cells; 3, some loss of crypts; 4, extensive loss of crypts); infiltration (0, none infiltration; 1, infiltration around crypt bases; 2, infiltration spreading to muscularis mucosa; 3, extensive infiltration in the muscularis mucosa with abundant oedema; 4, infiltration spreading to submucosa). The total score ranged from 0 to 8.

**Table 2:** Disease activity index score parameters (DAI).

Weight loss(%)	Stool	Bloody stool	Index
**0-1%**	Normal	None	0
**1-4%**	Soft and shaped	Between	1
**4-8%**	Loose	Slight	2
**8-12%**	Between	Between	3
**>12%**	Diarrhea	Gross bleeding	4

### MPO Activity Assay

Inflammatory cell (polymorphonuclear neutrophil) infiltration into colonic tissue was quantified by measuring MPO activity with an MPO assay kit (Nanjing Jiancheng Bio-engineering Institute, China), following the manufacturer’s instructions. MPO activity was expressed as units per gram of total protein (U/g) (20).

### Flow Cytometry Staining

The stimulated macrophages were preincubated with Fc Block (anti-CD16/CD32, BD Biosciences) for 10 min at 4°C. These cells were stained with PE-conjugated mAbs to CD11c (Biolegend). Macrophages were fixed, permeabilized using a FIX/PERM set (Biolegend) for 10 min at room temperature prior to intracellular staining with PE-anti-CD206 (BD Biosciences). Splenocytes were separated from spleens of mice and cultured in the medium above for 36 h. 2 µL/ml stimulation cocktail (Thermo Invitrogen, USA) was added to the RPMI 1640 medium for the last 10 h, and 0.66 μL/ml Golgistop™ (BD Biosciences) for the last 5 h. Cells were first preincubated with Fc Block for 30 min and stained for PerCP-Cy5.5-anti-CD3 and FITC-anti-CD4 antibodies (BD Biosciences) for 35 min at 4°C in the dark. Then cells were fixed, permeabilized using a FIX/PERM set (Biolegend) for 10 min at room temperature prior to intracellular staining with PE-anti-IL-4, APC-anti- IFN-γ antibodies or PE-anti-IL-10. Samples were analyzed by using a BD FACS Calibur flow cytometer and FlowJo software (Tree star Inc, Ashland, OR).

### Determination of Colon Cytokines

Cytokine levels of IFN-γ, IL-4 or IL-10 in the colon were determined as described previously (8). Briefly, a segment of the colon was excised and washed twice in clean PBS containing penicillin and streptomycin. Then, the colon was further cut into 1 cm^2^ pieces and placed in 24-well flat bottom-well culture plates with 1 ml RPMI 1640 supplemented with 100 U/ml penicillin and 100 μg/ml streptomycin at 37 °C, 5% CO_2_ for 24 h. The supernatant was collected, and cellular debris was removed by centrifugation. The levels of cytokines (IFN-γ, IL-4 or IL-10) in the supernatant were quantified by ELISA.

### Statistical Analysis

All results are expressed as the mean ± SD. Statistical analysis was performed using the GraphPad Prism 8 software for Windows. One-way, two-way analysis of variance (ANOVA) and independent exponent t test were used to compare the means and determine statistically significant differences between different conditions. p values are expressed as *p<0.05, **p< 0.01 and ***p<0.001.

## Results

### Nrf2 Was Involved in M2 Polarization Induced by *T. spiralis* ES *In Vitro*

**Figure 2:**
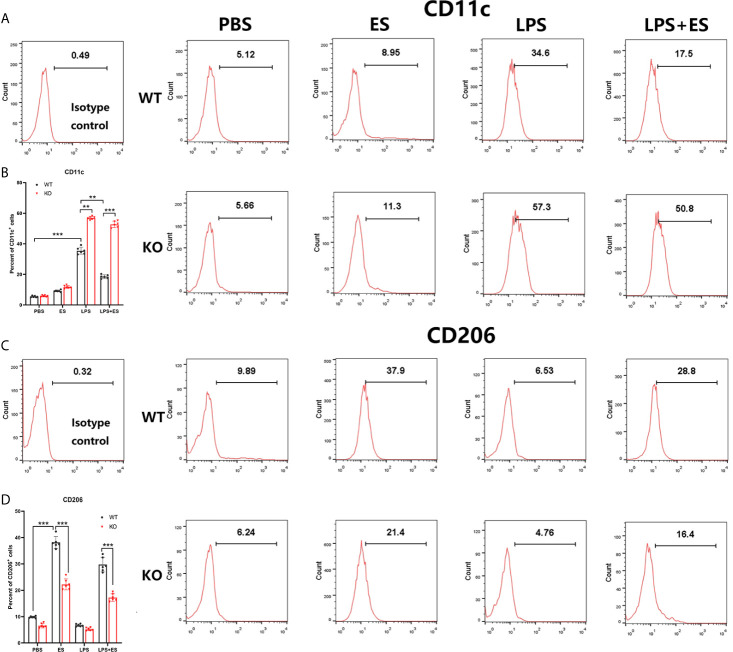
Expression of CD11c (M1 marker) and CD206 (M2 marker) on wild type (WT) or Nrf2 KO macrophages induced by *T. spiralis* ES. The stimulated macrophages were stained PE-conjugated mAbs to CD11c **(A, B)** or CD206 **(C, D)**, and analyzed by flow cytometry. Data are showed by the mean ± SD of each group (n=6) **P < 0.01, ***P < 0.001, ns, no significance, as indicated by the line (one-way ANOVA with Tukey’s *post hoc* test).

**Figure 3:**
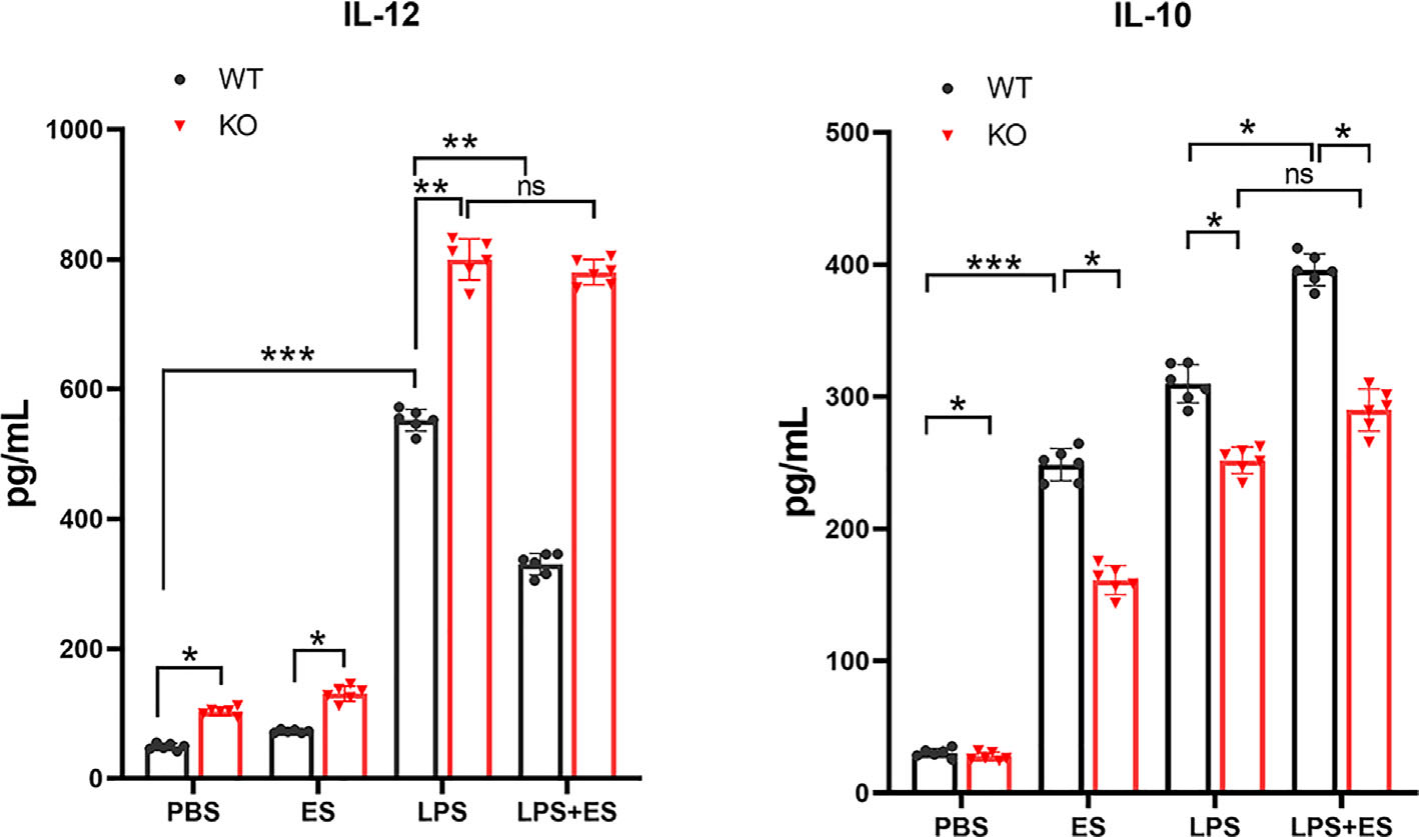
The levels of IL-12 and IL-10 secreted from wild type (WT) or Nrf2 KO macrophages induced by *T. spiralis* ES. Macrophages were enriched by positive selection with anti- F4/80 magnetic beads. These cells were stimulated with sterile PBS, ES or LPS alone for 24 h. And macrophages were pre-treated with ES (50 μg/ml) for 24 h before stimulation with LPS (100 ng/ml) for 24 h. Cell culture supernatants were collected and stored at −80°C. IL-12 and IL-10 levels in the supernatant were quantified by ELISA. Results are presented as the mean ± SD (n=6). *P < 0.05, **P < 0.01, ***P < 0.001, ns, no significance, as indicated by line (one-way ANOVA with Tukey’s *post hoc* test).

To investigate whether Nrf2 is involved in M2 polarization induced by *T. spiralis* ES, we compared the polarization of ES–treated macrophages from wild type (WT) mice and Nrf2 KO mice. Our results showed that ES increased the expression of Arginase-1 (Arg1) in WT macrophages but the enhanced expression of Arg1 and CD206 (M2 markers) induced by ES was decreased in Nrf2 KO macrophages (**Figures 1B**, **2C, D**). We found that ES inhibited LPS –induced high level of iNOS and CD11c (M1 markers) in WT macrophages while there was no significant difference between LPS group and LPS + ES group in Nrf2 KO macrophages (**Figures 1C**, **2A, B**). M1 is characterized by the expression of high levels of pro-inflammatory cytokines such as IL-12 (21). LPS can stimulate the level of pro-inflammatory cytokine IL-12, which was significantly decreased by ES in WT macrophages, but not Nrf2 KO macrophages (**Figure 3**). In contrast, M2 function in resolving inflammation and is characterized by the expression of anti-inflammatory cytokine IL-10 (22). We observed that both ES and LPS enhanced the level of IL-10, and combination of ES and LPS significantly increased IL-10 level in macrophages compared to PBS, ES or LPS group. However, either ES or LPS –induced level of IL-10 in Nrf2 KO macrophages was not as high as those in WT macrophages (**Figure 3**).

### ES –Treated Nrf2 KO Macrophages Failed to Alleviate the Severity of the Colitis in Mice *In Vivo*

**Figure 4:**
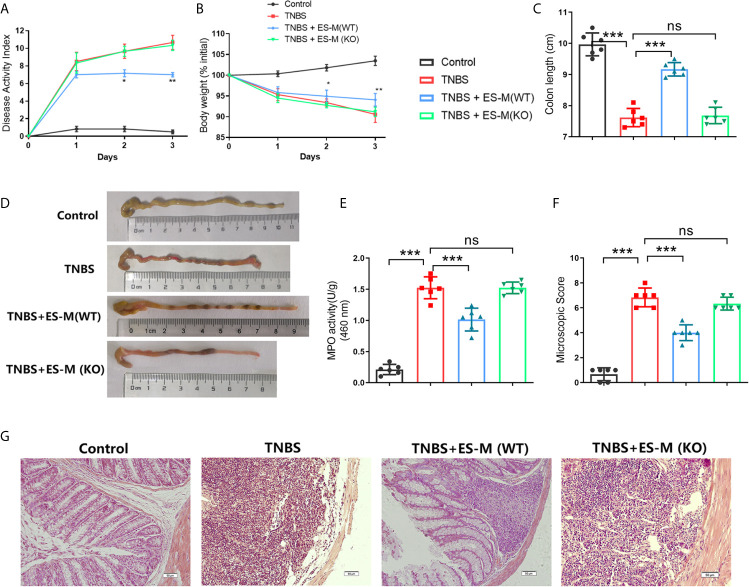
Effect of *T. spiralis* ES –induced wild type (WT) or Nrf2 KO macrophages (ES-M) on TNBS-induced colitis. For adoptive transfer, WT or Nrf2 KO macrophages induced by *T. spiralis* ES were washed (x3) with sterile PBS, and 1 × 10^6^ cells in 500 μL of sterile PBS were injected intravenously (i.v.) after TNBS challenge. Three days later, colitis was induced by TNBS in these recipient mice. Mice were humanely euthanized by CO_2,_ and then the colon and spleen were collected for the following experiments. The protective efficacy against TNBS challenge was determined in three independent experiments. One representative experiment is shown here. **(A)** Disease activity index (DAI) was measured during the disease process. **(B)** The daily mean weight change in each group was calculated. **(C, D)** After 3 days, colons were removed, and the lengths of their colons were measured and recorded. **(E)** Myeloperoxidase (MPO) activity in the colonic tissues was detected. The colonic segments were stained with hematoxylin and eosin (H.E.) staining according to standard protocols. **(F)** The colons from each experimental group (n=6) were processed for histological evaluation (200×). **(G)** Histopathological damage scores were determined for the colon tissue samples. The results are representative of at least three independent experiments and expressed as the mean ± SD of each group (n=6). ***P < 0.001, ns, no significance, as indicated by line (one-way ANOVA with Tukey’s posttest) on the same day.

To evaluate the therapeutic effect of ES –treated macrophage (ES -M) on colitis, we established an acute model of colitis in mice and performed adoptive transfer of these macrophages. We observed that DAI scores of the TNBS group were significantly elevated 3 days after induction (**Figure 4A**). While adoptive transfer of ES –treated WT macrophages decreased the DAI scores, ES –treated Nrf2 KO macrophage (ES -M (KO)) did not reduce these scores. The weight loss rate of TNBS-induced mice after 3 days was close to 10%. Mice treated with ES-M after TNBS induction showed significant weight loss on day 2 and day 3 (**Figure 4B**). The TNBS group (7.617 ± 0.2927 cm) and TNBS + ES -M (KO) (7.683 ± 0.2639 cm) group showed colonic shortening, whereas colon length the ES -M group (9.167 ± 0.2160 cm) was not shortened compared to TNBS group (**Figures 4C, D**). In addition, treatment of ES -M, but not ES -M (KO), significantly decreased the level of MPO induced by TNBS (TNBS: 1.499 ± 0.216 U/g; ES -M +TNBS: 0.9375 ± 0.1797 U/g; ES -M (KO) +TNBS: 1.465 ± 0.1248 U/g) (**Figure 4E**). Histopathological injury in the colon was further measured. We observed that the TNBS group showed distortion of the crypts and extensive cellular infiltration. Administration of ES -M obviously improved the pathological injury, whereas ES -M (KO) failed to alleviate the pathological injury (**Figure 4F, G**). These results indicated that the treatment of ES -M alleviated the severity of inflammation in the colon. However, no significant difference between TNBS group and TNBS + ES -M (KO) group was evident.

### Nrf2 of Macrophages Played a Critical Role in Regulating Cytokine Production Induced by Adoptive Transfer *In Vivo*

**Figure 5:**
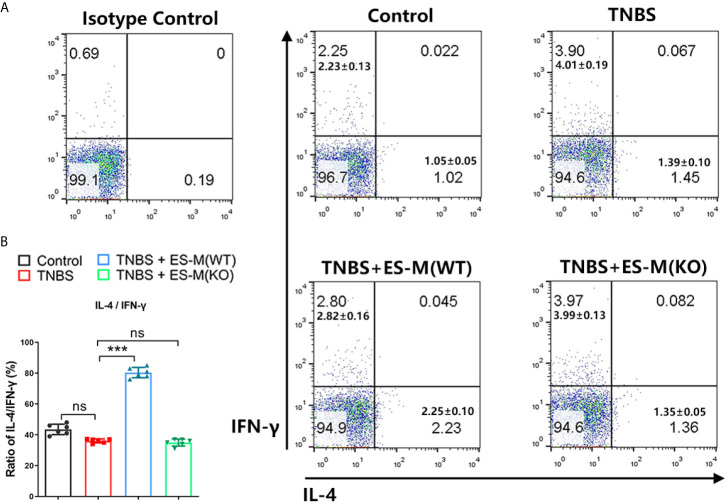
Differentiation of CD3^+^ CD4^+^ IFN-γ^+^ or IL-4^+^T cells of *T. spiralis* ES –induced wild type (WT) or Nrf2 KO macrophages (ES-M) on TNBS-induced colitis. **(A)** CD3^+^ CD4^+^ T cells were gated. IFN-γ^+^, IL-4^+^ T cells populations were determined. **(B)** The ratio of IL-4/IFN-γ was shown. Data are shown as the means ± SD (three independent experiments) of each group (n=6). ***p < 0.001, ns, no significance, as indicated by line (one-way ANOVA with Tukey’s posttest). These figures are representative of three independent experiments.

**Figure 6:**
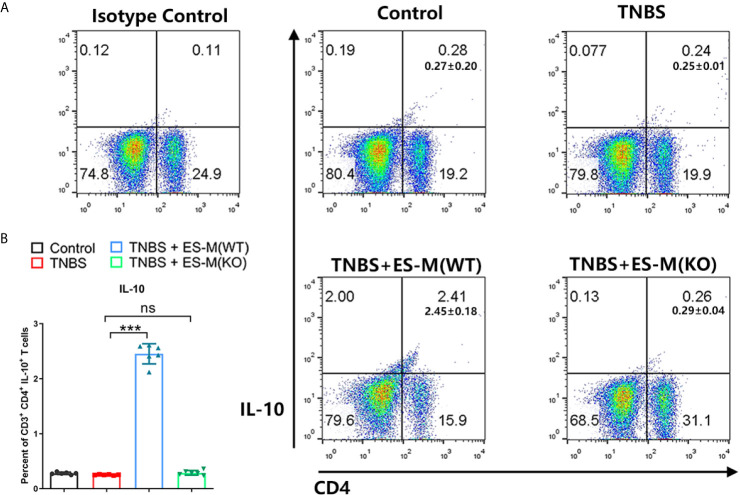
Differentiation of CD3^+^ CD4^+^ IL-10^+^T cells of *T. spiralis* ES –induced wild type (WT) or Nrf2 KO macrophages (ES-M) on TNBS-induced colitis. **(A)** CD3^+^ T cells were gated. CD4^+^IL-10^+^ T cells populations were determined. **(B)** The percent of CD4^+^IL-10^+^ T cells were shown. Data are shown as the means ± SD (three independent experiments) of each group (n=6). ***p < 0.001, ns, no significance, as indicated by line (one-way ANOVA with Tukey’s posttest). These figures are representative of three independent experiments.

To assess the induction of Th1 and Th2 immune response, the splenocytes were isolated and assessed by flow cytometry. Compared to the control group, the CD3^+^ CD4^+^ IFN-γ^+^ T cell population associated with Th1 immune response in TNBS-induced mice was significantly enhanced (**Figure 5A**). Compared to the TNBS group, Th1 cells in the ES-M instead of ES-M (KO) treatment group were significantly reduced, and the number of CD3^+^ CD4^+^ IL-4^+^ T cells was significantly increased, which were defined as Th2 cells (**Figure 5A**). The ratio (percentage of IL-4/percentage of IFN-γ) in ES -M group (80.33 ± 3.327%) was higher than in the TNBS group (36.03 ± 1.291%) and ES -M (KO) group (34.98 ± 2.263%) (**Figure 5B**). IL-10 is essential for the anti-colitis effect (23). Further, we found the population of CD3^+^ CD4^+^ IL-10^+^ T cells was increased in ES -M group rather than in ES -M (KO) group (**Figure 6**).

**Figure 7:**
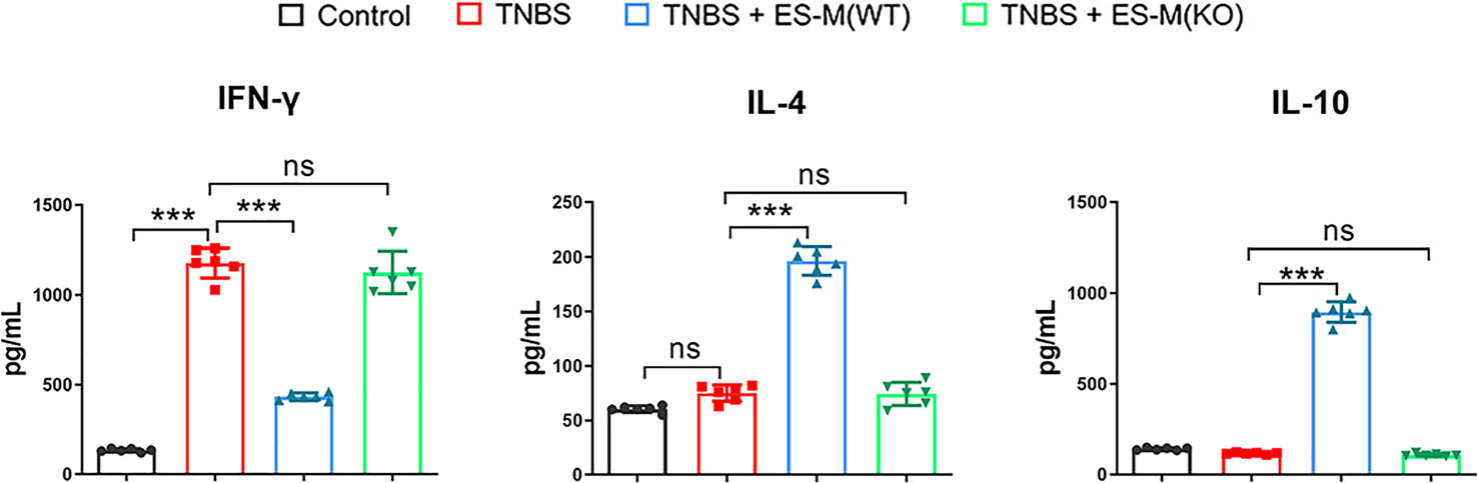
Cytokine production in colons of *T. spiralis* ES –induced wild type (WT) or Nrf2 KO macrophages (ES-M) on TNBS-induced colitis. The colon culture supernatant was used to determine the cytokine production. IFN-γ, IL-4 and IL-10 levels were measured by ELISA. Data are shown as the means ± SD (three independent experiments) of each group (n=6). ***p < 0.001, ns, no significance, as indicated by line (one-way ANOVA with Tukey’s posttest).

In addition, cytokine production in the culture supernatant of colon tissue was determined. The level of IFN-γ in the colon was significantly elevated in the mice from TNBS group (1178.0 ± 82.80 pg/ml) and ES -M (KO) + TNBS group (1127.1 ± 117.79 pg/ml), but was significantly inhibited in the ES -M -treated mice (433.7 ± 21.35 pg/ml). There was no effect of TNBS alone on IL-4 production (75.1 ± 7.51 pg/ml), and ES -M -treated group had significantly higher level of IL-4 (196.3 ± 13.02 pg/ml), which is suggested to be associated with the Th2 immune response. ES -M -treated group also exhibited higher level of IL-10 than TNBS group (TNBS + ES -M: 896.3 ± 56.30 pg/ml; TNBS: 117.0 ± 4.858 pg/ml). However, treatment of ES -M (KO) did not enhance the levels of IL-4 (74.33 ± 10.65 pg/ml) and IL-10 (108.3 ± 7.367 pg/ml) (**Figure 7**).

## Discussion

Inflammatory bowel disease (IBD), including the two main clinical entities, Crohn’s disease (CD) and ulcerative colitis (UC), are chronic relapsing disorders of the gastrointestinal tract (24). Crohn’s disease (CD) is a chronic dysregulated inflammatory disease of intestinal tract. Several studies have demonstrated the therapeutic potential of helminths in the treatment of colitis (25–27). Previously, we have demonstrated that infection of *Trichinella spiralis* reduce the severity of TNBS –induced colitis (28). However, helminth therapy is hard to accept for patients because of many ethical issues that might be raised. In the present study, we aimed to investigate a potentially new approach in the treatment of CD.

Cellular immunotherapy has been emerging as a therapeutic option, and macrophages play central roles in the development of colitis. Helminths and their products promote macrophage differentiation into alternatively activated macrophages (M2) that control the Th1 and upregulate the Th2 immune response (5). It has been reported that helminths protect from colitis through induction of alternatively activated macrophages (M2) (4). Macrophages treated with antigen from helminth can protect against colitis (9, 29, 30). A recent study (9) has found that adoptive transfer of *T. spiralis* excretory/secretory (ES) -treated macrophages inhibited dextran sulfate sodium (DSS) -induced UC. However, current evidence suggests that UC is a modified T-helper-2 (Th2) disease, while CD is Th1 driven (31). In this study, we investigated the effect of *T. spiralis* ES –treated macrophage (ES -M) on TNBS –induced colitis, which is a well-established model of human CD (32). Our results showed that adoptive transfer of ES -M attenuated the inflammation of TNBS-induced colitis through inhibiting Th1 –related cytokine IFN-γ. The mice treated with ES -M exhibited increased levels of IL-4 and IL-10 *in vivo*. IL-4 is produced by Th2 cells and can suppress pro-inflammatory cytokine and alleviate colitis (33). Th2 cytokines can promote the development of goblet cells and mast cells, thereby changing the intestinal environment, especially IL-4 (34). IL-10 has been previously related to Th2 differentiation (35) and is known as an anti-inflammatory cytokines (36). Notably, IL-10 administration have proven efficacy in suppressing colitis in mice (4).

Consistent with this previous study (9), we also found that *T. spiralis* ES induced M2 *in vitro*. Macrophages can change their phenotype in response to many different stimuli, a process called activation. The classically activated or inflammatory macrophages (M1) is characterized by the expression of high levels of pro-inflammatory factor such as inducible nitric oxide synthase (iNOS), CD11c and IL-12 (21). In contrast, M2 function in resolving inflammation and are characterized by the levels of anti-inflammatory cytokine IL-10, CD206 and the arginase-1 (Arg-1) (22). We observed that treatment with ES were able to inhibit the increase in M1 markers expression induced by LPS and enhance the M2 markers expression, indicating that *T. spiralis* ES could shift the phenotype from M1 to M2. However, the mechanism by which *T. spiralis* ES polarizes macrophages remains elusive.

Notably, increasing colonic inflammation is associated with oxidative stress injury (22). We demonstrated that TNBS –induced mice exhibited high level of MPO activity. Thus, we speculated that *T. spiralis* ES participate in regulating oxidative stress in macrophages. Nrf2 (nuclear factor E2-related factor 2) has been known for over 10 years, to be the key transcription factor regulating the antioxidant response which is crucial for cytoprotection against extracellular stresses. Nrf2 positively regulates transcription of genes having antioxidant effects (37). Nrf2 expression and activation occurs during the initial contact of some *Leishmania* species with cells, increasing the amount of gene products associated with M2 macrophage characteristics (38). We also showed that Nrf2 may be involved in altering the phenotype of ES –induced macrophages. Our results are in agreement with a recent study suggesting that *T. spiralis* infection can mitigate inflammation by upregulating Nrf2 expression (16). We found that ES -treated Nrf2 KO macrophages expressed less IL-10, which is essential for the anti- inflammatory effect (23) and a higher level of IL-12 which can promote a Th1 response (39). The capacity of ES to reduce LPS -induced inflammation when lacking Nrf2 was impaired, suggesting that anti-inflammatory role of ES may be primarily involved in the Nrf2 pathway. It has been also reported that *T. gondii* can balance the oxidative stress of IFN-γ and TNF-α –stimulated macropahges (40). In addition, we not only showed that Nrf2 participated in the development of *T. spiralis* ES -induced M2, but also verified that Nrf2 KO macrophage induced by *T. spiralis* ES failed to attenuate the severity of colitis and produced lower levels of IL-10 *in vivo*, suggesting that Nrf2 KO macrophages treated by ES did not exert their anti-inflammatory effects on colitis. It has been also reported that Nrf2 results in the production of IL-10 (41).

Moreover, there are many mechanisms that come into play, making the Nrf2 control system very complex. Nrf2 is regulated by microRNAs, including miR-200a (42). It will also be interesting to explore the role of microRNA from *T. spiralis* in activation of Nrf2. Previously, we provided evidence for the presence of microRNA in different development stages of *T. spiralis*, suggesting a potential regulatory function in the host (43, 44). There are helminth-specific miRNAs in extracellular vesicles (EVs) isolated from *T. spiralis* ES with immunomodulatory potential (20). Prediction of the interactions between miRNAs of *Ts*-EVs and murine host genes indicates that Nrf2 may be the target regulated by some miRNAs. Further studies are warranted to discovery new miRNA targeting Nrf2. In addition, a protein kinase has recently been shown to have a role in activating Nrf2 (37). We also found several protein kinase families in a previous study (44). Interestingly, many genes encoding superoxide dismutase (SOD), glutathione perxidases and heat shock protein were identified in ML stages of *T. spiralis* (44), indicating that these antioxidants may play a role in improving antioxidant defenses in stress conditions. Identification of new microRNA or proteins involved in the Nrf2 in macrophages requires further studies.

Our findings demonstrated that treatment of *T. spiralis* ES -induced M2 alleviated the severity of TNBS-induced colitis in mice. Furthermore, we confirmed that Nrf2 participated in the development of *T. spiralis* ES -induced M2 *in vitro* and played a critical role in effect of ES -M on colitis *in vivo*. These results provide evidence that *T. spiralis* ES -induced M2 may serve as a potentially new approach for the treatment of CD or other Th1 immune mediated diseases.

## Data Availability Statement

The original contributions presented in the study are included in the article/supplementary materials. Further inquiries can be directed to the corresponding authors.

## Ethics Statement

All animal experiments were performed according to regulations of the Administration of Affairs Concerning Experimental Animals in China. The protocol was approved by the Institutional Animal Care and Use Committee of Jilin University (20170318).

## Author Contributions

XJ and XB conceived and designed the experiments. XJ, XB, YZ, ZD, and JP performed the experiments. XJ, XB, and XL analyzed the data. XJ, XB, and ML drafted the paper. YZ, ZD, JP, XL, and ML revised the paper. All authors contributed to the article and approved the submitted version.

## Funding

This study was supported by The National Key Research and Development Program of China (2018YFC1602500, 2017YFC1601200), the National Natural Science Foundation of China (31520103916, 31872467) and Program for JLU Science and Technology Innovative Research Team(2017TD-32).

## Conflict of Interest

The authors declare that the research was conducted in the absence of any commercial or financial relationships that could be construed as a potential conflict of interest.
